# Risk classification priorities in an emergency unit and outcomes of the service provided

**DOI:** 10.1590/1518-8345.2345.2974

**Published:** 2017-12-21

**Authors:** Rafael Silva Marconato, Maria Ines Monteiro

**Affiliations:** 1 Doctoral Student, Faculdade de Enfermagem, Universidade Estadual de Campinas, Campinas, SP, Brazil. RN, Hospital de Clínicas, Universidade Estadual de Campinas, Campinas, SP, Brazil.; 2 PhD, Associate Professor, Faculdade de Enfermagem, Universidade Estadual de Campinas, Campinas, SP , Brazil.

**Keywords:** Descriptors: Emergency Nursing, Emergency Medical Services, Emergency Identification, Triage, Protocols, Nursing

## Abstract

**Objective:**

to check the association of the proposed priorities of the institutional protocol of risk classification with the outcomes and evaluate the profile of the care provided in an emergency unit.

**Method:**

observational epidemiological study based on data from the computerized files of a Reference Emergency Unit. Care provided to adults was evaluated regarding risk classification and outcomes (death, hospitalization and hospital discharge) based on the information recorded in the emergency bulletin.

**Results:**

the mean age of the 97,099 registered patients was 43.4 years; 81.5% cases were spontaneous demand; 41.2% had been classified as green, 15.3% yellow, 3.7% blue, 3% red and 36.and 9% had not received a classification; 90.2% of the patients had been discharged, 9.4% hospitalized and 0.4% had died. Among patients who were discharged, 14.7% had been classified as yellow or red, 13.6% green or blue, and 1.8% as blue or green.

**Conclusion:**

the protocol of risk classification showed good sensitivity to predict serious situations that can progress to death or hospitalization.

## Introduction

Overcrowding of emergency services, defined as the situation in which attention to urgencies is compromised by the excessive demand in relation to the available resources, represents a relevant public health problem in several countries. Scholars devise strategies to reduce the known negative effects of these events, such as increased mortality, prolonged hospitalization time and increased readmissions. Patient evaluation by nurses using risk classification protocols represents an essential strategy to minimize these problems^1^.

In the last decades, protocols have been developed and published to assist nursing professionals in this evaluation. The best known are the *Manchester Triage System* (MTS), the Australian *Australasian Triage Scale* (ATS), the Canadian *Canadian Triage and Acuity Scale* (CTAS) and the American *Emergency Severity Index* (ESI)^2^. Studies in several countries have demonstrated the validity and effectiveness of these protocols as important tools for the organization of emergency services^3^ ^-^ ^6^.

In Brazil, the Ministry of Health launched in 2004 the Humanized SUS program with the objective of uniting managers, workers and users to make health services more humanized and efficient^7^.

After a few years, in 2009, the Booklet on Reception and Risk Classification in Emergency Services was published as a reference to the concepts of this program, guiding and inviting urgency services to create Reception Services with Risk Classification. The purpose would be to organize the entry door in the system, which is routinely overcrowded with demands that do not correspond to the complexity of the services offered^8^.

Following the guidelines proposed by the Ministry of Health in the Humanized SUS program, which determined that each unit should develop its own protocol according to the regional characteristics of the population and its attendance capacity, a master’s thesis developed and validated in 2010 an institutional protocol based on the population profile, the main complaints presented by users, and the flows in the emergency service of a large university hospital in the city of Campinas, São Paulo, Brazil^9^.

This protocol^9^ was adopted by the institution and served to classify patients on the basis of four degrees of complexity indicated by the colors: red, yellow, green and blue. Patients classified as red had the highest priority, following in this order until blue, which was considered as timely priority or of less complexity.

The protocol had 35 flowcharts and all the nurses working in the risk classification service had been trained to apply it. In the period from 2010 to 2017, this Emergency Unit applied this tool for risk assessment of users^9^.

The strategy of creating institutional protocols was also efficient in a research carried out in an emergency unit in São Paulo, Brazil, which used a protocol based on the expertise of its professionals and on the characteristics of its population. When the risk classification of patients was related to the outcomes death and hospital discharge within less than 24 hours, the authors demonstrated and efficacy consistent with other worldwide known protocols such as the MTS and the ESI^10^ ^-^ ^11^.

In this context, this study aimed to associate the service priorities proposed by the institutional protocol with the outcomes of the care provided in the emergency unit and its ability to predict patient severity, as well as to evaluate the profile of the care provided in the emergency unit.

## Method

Epidemiological observational study based on data from the computerized medical files of the Referral Emergency Unit of the Clinical Hospital of the State University of Campinas, Campinas, São Paulo, Brazil.

The study population corresponded to all the adults who received care, as registered in the Emergency Bulletin, at the study site between January 1 and December 31, 2014. Patients aged 14 years and over were included in the study; users aged 14 to 18 follow the same care processes provided to adults. Patients under 14 years of age were excluded because in this unit they are considered pediatric patients and have a differentiated flow of reception and medical care.

We decided not to differentiate the medical referral specialties after risk classification because this is a general hospital and therefore receives patients from neurosurgery and medical, surgical, neurological, psychiatric, ophthalmological and orthopedic clinics. Patients in these last three specialties have the care registered, but they are not always referred to risk classification. The pediatric area was excluded. Gynecological care takes place in a specialized center at the institution studied, which is not part of the Emergency Unit.

The risk classification received in the first assistance provided by nurses and the outcome - death, hospitalization and hospital discharge - were evaluated.

Data were obtained in the hospital system, in which an administrative professional collects and inserts identification information in the computerized system: name, age, address, skin color, if a work accident happened in that particular case, and if there was a referral or spontaneous demand, resulting in the Urgent Care Bulletin.

The Urgent Care Bulletin is then printed and the patient or the companion checks and signs the validation and consent of the recorded data. The form is sent to the nurse who proceeds to the evaluation and risk classification, with later medical care and directing of the conducts, according to the priority. Records coming from the risk classification and the service are hand written in this same form and, after the service, they return to reception and the administrative professional registers the risk classification (blue, green, yellow and red) and the outcome (death, hospitalization or hospital discharge). These data are recorded in the hospital database and exported into an Excel® spreadsheet, which is the data source of this research.

To perform the analysis, the population was stratified into six groups according to the risk classification: red, yellow, green or blue, those assisted without risk classification and losses. There was also a redistribution of the total number of assistances into two other groups according to the complexity of the situation: serious - grouping the red and yellow classification and, non-serious - green and blue.

These subgroups were compared as for the outcomes (death, hospitalization or hospital discharge), and associated to: age group, divided into five categories - 14 to 17; 18 to 29; 30 to 59; 60 to 79; and 80 years or more; length of stay in the unit - less than 24 hours; from one to four days; and five days or more; and time of arrival at the unit - from 7:00 to 12:59; 13:00 to 18:59; 19:00 to 00:59; and 1:00 to 6:59. 

The chi-square and Kruskal-Wallis tests were applied for the relationship with age, using the SAS® software and considering a statistical significance level of 5.0%.

The research project respected the Declaration of Helsinki and the Resolution 466/12, and was approved by the Research Ethics Committee, CAAE 68244317.3.0000.5404, via Brazil Platform, and had no need of Informed Consent Forms (ICF) because this is a documentary research.

## Results

Data from 97,099 consultations were analyzed; the mean age of the individuals was 43.4 years (Standard Deviation SD = 8.8), with a minimum of 14 and a maximum of 106 years. A total of 71,907 (74.3%) patients remained in the Emergency Unit less than one day, 79,133 (81.5%) came by spontaneous demand and 78,175 (90.2%) had hospital discharge as outcome. According to the risk classification, 14,791 (15.3%) patients were classified as yellow and 43,307 (44.8%) as non-serious complexity patients, according to Table 1.

**Table 1:** Characterization of the consultations in the reference emergency unit. Campinas, SP, Brazil, 2014

**Variables**		**n**	**%**
**Age group**			
	**14 - 17**	**4,553**	**4.7**
	**18 - 29**	**24,431**	**25.2**
	**30 - 59**	**46,499**	**47.9**
	**60 - 79**	**18,285**	**18.8**
	**80 or more**	**3,329**	**3.4**
	**Total**	**97,097**	**100.0**
**Length of stay**			
	**< 1 day**	**71,907**	**74.3**
	**1 to 4 days**	**24,170**	**25.0**
	**5 or more days**	**711**	**0.7**
	**Total**	**96,788**	**100.0**
**Source**			
	**Spontaneous demand**	**79,133**	**81.5**
	**Transfer from another service**	**7,596**	**7.8**
	**Return for revaluation**	**6,371**	**6.6**
	**Elective hospitalization**	**1,479**	**1.5**
	**Pre-hospital care services**	**917**	**0.9**
	**Others**	**1,553**	**1.6**
	**Total**	**97,049**	**100.0**
**Risk classification**			
	**Without classification**	**35,653**	**36.9**
	**Red**	**2,959**	**3.1**
	**Yellow**	**14,791**	**15.3**
	**Green**	**39,757**	**41.1**
	**Blue**	**3,550**	**3.7**
	**Total**	**96,710**	**100.0**
**Categorized risk classification**			
	**Without classification**	**35,653**	**36.9**
	**Serious (red and yellow)**	**17,750**	**18.4**
	**Non-serious (green and blue)**	**43,307**	**44.8**
	**Total**	**96,710**	**100.00**
**Outcome**			
	**Discharge**	**78,175**	**90.2**
	**Hospitalization**	**8,186**	**9.4**
	**Death**	**334**	**0.4**
	**Total**	**86,695**	**100.0**

**Figure 1:**
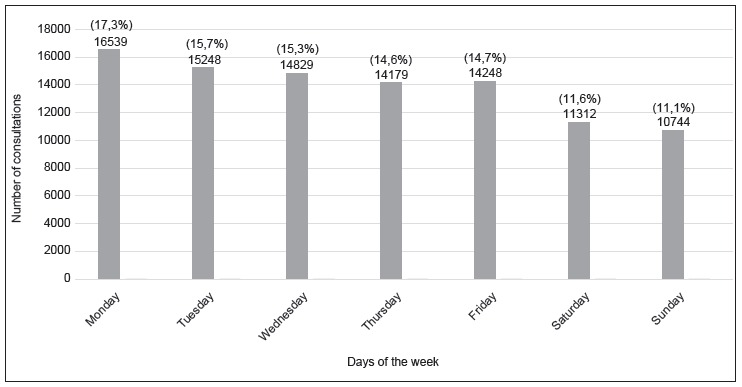
Distribution of the opening frequency of the Emergency Bulletin according to days of the week. Campinas, SP, Brazil, 2014

**Figure 2:**
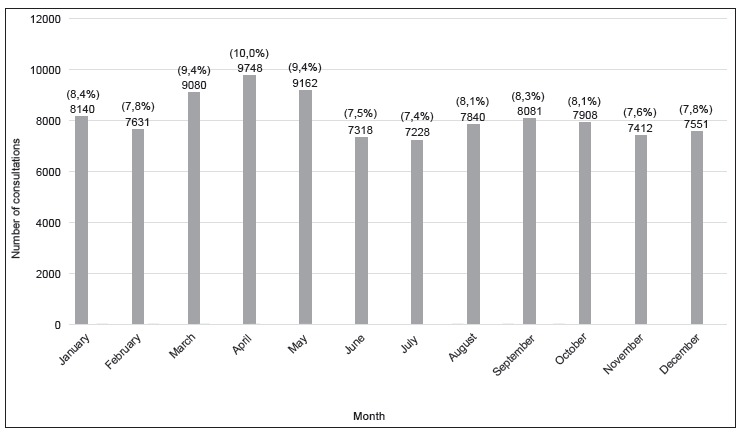
Distribution of the opening frequency of the Emergency Bulletin according to months. Campinas, SP, Brazil, 2014

Figures 1 and 2 present the opening frequency of the Emergency Bulletin per day of the week and month of 2014, with decrease at weekends and greater number of consultations in the months of March, April and May.

Table 2 shows the associations between the risk classification assigned by nurses at the patient’s arrival and the variables: service outcome, age group, length of stay and arrival time. All associations had a statistically significant difference (p < 0.001 - Chi-square test).

**Table 2:** Presentation of risk classification according to outcome, age group, length of stay and arrival time. Campinas, SP, Brazil, 2014

**Clasificación de riesgo**											
	**Without classification**		**Red**		**Yellow**		**Green**		**Blue**		**Total 100%**
	**n(%)**		**N(%)**		**N(%)**		**N(%)**		**N(%)**		**N**
**Outcome**											
**Discharge**	**25937(33,2)**		**1356(1,7)**		**12927(16,5)**		**35700(45,7)**		**2221(2,8)**		**78141**
**Hospitalization**	**4262(52,1)**		**1353(16,5)**		**1425(17,4)**		**1111(13,6)**		**31(0,4)**		**8182**
**Death**	**105(31,4)**		**172(51,5)**		**51(15,3)**		**05(1,5)**		**01(0,3)**		**334**
**Not informed**	**00(0,0)**		**00(0,0)**		**00(0,0)**		**00(0,0)**		**00(0,0)**		**10442**
**Age group (years)**											
**14 - 17**	**1645(36,2)**		**89(2,0)**		**588(13,0)**		**2030(44,7)**		**189(0,2)**		**4541**
**18 - 29**	**9086(37,3)**		**511(2,1)**		**2808(11,5)**		**10914(44,8)**		**1028(4,2)**		**24347**
**30 - 59**	**17191(37,1)**		**1275(2,8)**		**6492(14,0)**		**19192(41,4)**		**2167(4,7)**		**46317**
**60 - 79**	**6632(36,5)**		**836(4,6)**		**3943(21,7)**		**6618(36,4)**		**157(0,9)**		**18186**
**80 or more**	**1098(33,1)**		**248(7,5)**		**960(28,9)**		**1003(30,2)**		**08(0,2)**		**3317**
**Not informed**	**00(0,0)**		**00(0,0)**		**00(0,0)**		**00(0,0)**		**00(0,0)**		**391**
**Length of stay**											
**< 1 day**	**23840(33,2)**		**1359(1,9)**		**10572(14,7)**		**32920(45,8)**		**3186(4,4)**		**71877**
**1 to 4 days**	**11311(46,9)**		**1484(6,2)**		**4157(17,2)**		**6792(28,2)**		**363(1,5)**		**24107**
**5 or more days**	**486(68,6)**		**115(16,2)**		**62(8,7)**		**45(6,4)**		**01(0,1)**		**709**
**Not informed**	**00(0,0)**		**00(0,0)**		**00(0,0)**		**00(0,0)**		**00(0,0)**		**406**
**Arrival time**											
**7:00 to 12:59**	**15117(37,4)**		**1064(2,6)**		**5514(13,6)**		**16638(41,2)**		**2067(5,1)**		**40400**
**13:00 to 18:59**	**11536(37,9)**		**927(3,0)**		**5341(17,6)**		**11705(38,5)**		**901(3,0)**		**30410**
**19:00 to 00:59**	**5332(30,4)**		**533(3,0)**		**2987(17,0)**		**8350(47,5)**		**358(2,0)**		**17560**
**1:00 to 6:59**	**3668(44,0)**		**435(5,2)**		**949(11,4)**		**3064(36,7)**		**224(2,7)**		**8340**
**Not informed**	**00(0,0)**		**00(0,0)**		**00(0,0)**		**00(0,0)**		**00(0,0)**		**389**

Table 3 shows the relationship between risk classification categories and patient age. The associations showed statistically significant difference (p < 0.001 - Kruskal-Wallis test).

**Table 3:** Descriptive analysis of risk classification categorized according to patient age. Campinas, SP, Brazil, 2014

	**Category**	**n**	**Mean**	**Standard deviation**	**Minimum**	Q1*	**Median**	**Q3** ^**†**^	**Maximum**
**Edad**	**Without classification**	**35652**	**43,1**	**18,6**	**14**	**27**	**41**	**57**	**105**
	**Serious**	**17750**	**49,0**	**20,2**	**14**	**31**	**49**	**65**	**106**
	**Non-serious**	**43306**	**41,3**	**18,0**	**14**	**26**	**39**	**54**	**103**
	**Without classification**	**35652**	**43,1**	**18,6**	**14**	**27**	**41**	**57**	**105**
	**Red**	**2959**	**50,6**	**20,4**	**14**	**33**	**51**	**67**	**104**
	**Yellow**	**14791**	**48,7**	**20,1**	**14**	**31**	**49**	**65**	**106**
	**Green**	**39757**	**41,7**	**18,2**	**14**	**26**	**39**	**55**	**103**
	**Blue**	**3549**	**37,7**	**14,1**	**14**	**25**	**37**	**49**	**93**

* First quartile (Q1) / † Third quartile (Q3)

## Discussion

The age of the participants averaged 43.4 years (SD ± 18.8). Among them, 21,614 (22.3%) were aged 60 years or more, of which 3,329 (3.5%) were 80 years or older. These values ​​are close to the current profile of the Brazilian population, whose predominance of adults, with an accelerated and exponential increase of the elderly, especially of octogenarian people. Increasing life expectancy requires a differentiated look towards the health care of aging people, also in emergency services.

This part of the population needs special attention when it comes to risk classification because they present a greater complexity and risk of complications. The prevalence of death of patients over 60 years of age was 202, representing 63.0% of all deaths occurred in the unit. A multicenter study evaluating emergency services in the Netherlands and Portugal to verify the validity of the Manchester System for Risk Classification underscored the importance of a more accurate and attentive assessment of vulnerable populations such as children and the elderly^10^.

The distribution of the search for the unit had a small variation throughout the months of the year, with an average of 8,091 users per month, with emphasis to an increase in the months from March to May caused by a dengue epidemic that occurred in Campinas in 2014^12^ ^-^ ^14^, as well as the increase in the incidence of respiratory cases in the coolest months in Brazil.

The high number of patients without classification - 35,653 (36.9%) - is explained by some routines of the Reference Emergency Unit such as elective hospitalizations, returns or patients directly referred to medical specialties that have no need of risk classification. Another common situation is the arrival of patients in situation of frank emergency such as those with firearm-related injuries, for example, who are sent to the emergency room before being evaluated. The later may justify, in large part, the high number of deaths among the non-classified cases, since this is a reference hospital in the region.

 There was a predominance of the green category in the risk classifications, i.e. the one that indicates less complexity, 39,757 (41.1%), followed by the blue and green categories, i.e. low priority patients looking for the unit, 43,307 (44.8%). These results are ​​similar to those shown in studies developed in units that use institutional protocols to classify patients according to five categories of priority: red, orange, yellow, green and blue^15^, such as the Federal School Hospital, in the city of São Paulo, Brazil, in which 73.7% of the patients had been classified as green or blue. Another study developed in the city of São Paulo found that 61.0% of the patients who seek the unit had received the green classification^16^.

Studies that evaluated the percentage of classified people using internationally recognized protocols such as the Manchester protocol showed the same trend. A Portuguese institution^5^ found that 72.9% of the users had been classified as being in situations of low priority, emphasizing that in the case of this protocol, low priority was indicated by the yellow color. Data from a study carried out in a German hospital using the Manchester System showed that the sum of the first three categories of low priority in the service totaled 80.0% of the patients^17^.

In this perspective, the data analyzed in the present Reference Emergency Unit are in accordance with the reality and may reflect a misuse of emergency services by the population, because people seek these services in situations that are not emergencies, but as the only access to health care, disregarding the primary care, which should largely absorb this low complexity demand.

American study investigated the reasons why users sought emergency services and the main justifications found were: difficulty of scheduling an outpatient consultation, or lack of knowledge of the existence of this service; the idea that the health problem could not wait any longer; and the idea that emergency services provide better quality services^18^.

The examination of the search for service per day of the week also showed a decrease during the week; the highest demand was seen on Mondays, 16,539 (17.0%), and the lowest demand on Sundays, 10,744 (11.0%). This trend again demonstrates that many users seek the service without a factual emergency. Their health condition allows them to wait for the day of the week to seek professional help.

The opening times in the consultation tickets also point to this trend, since most of the 40,640 tickets (41.8%) were delivered in the morning shift. The data related to the day of the week and the period corroborated a study^19^ conducted in 2011 in the same unit, in which 89.0% of the tickets had been distributed during the morning, and Mondays reunited 17.0% of the visits, while Saturdays and Sundays, 11.0% and 10.0%, respectively. This shows that this profile persists after almost a decade.

Regarding the outcome of patients after medical care, the majority were discharged, 90.17% (78,175). Another important aspect to be highlighted is that most patients remained less than 24 hours in the unit. These data differ from those found in a study carried out in the same unit in 2008, in which only 74.1% of patients had received hospital discharge^19^.

Other emergency units showed a similar tendency in relation to the outcome of patients after care, including those of a Portuguese hospital^17^, in which more than 90.0% had received hospital discharge, and the emergency service in São Paulo, Brazil, where 94.5% of the patients had been discharged, 4.3% had been hospitalized and 1.2% had died. All identified deaths were classified as priority by the institutional risk classification protocol of the respective unit, evidencing the sensitivity of the instrument^16^.

In the present study, the number of patients who died in the unit in 2014 was 334 (0.3%). When this data was correlated with the risk classification upon arrival, it was observed that deaths occurred in 66.7% of the patients classified as being in serious situation (red and yellow), whereas only 1.7% deaths happened in the low priority group (green and blue). There was also an expressive number (31.4%) of patients who died and who had not passed through risk classification, which is explained by the fact that it is common for patients admitted in very serious situations to be promptly forwarded to the emergency room before classification. However, these data demonstrate the adequate sensitivity of the institutional protocol studied in predicting gravity situations.

A study carried out in a Portuguese emergency service using the Manchester system for risk classification found that, when classified as a high priority, the risk of death was 5.58 times greater than that of patients with low priority of medical service^20^.

## Conclusion

The data on the medical service performed at the Reference Emergency Unit corroborate the reality of similar services in Brazil and worldwide, with a high sensitivity of the risk classifications in relation to the outcome of the medical service and provides evidence of the need for reorganization of health systems in order to increase the resilience of primary care services and decrease the number of people seeking emergency services for the wrong purposes.

The results obtained here have limitations, since the data were retroactively and secondarily extracted, and therefore allow for a divergence between the reality presented and the one identified in the data. The risk classification protocol studied here showed good sensitivity to predict serious situations that can progress to death or hospitalization; this protocol can be used as a tool in emergency services to increase the safety of patients who seek them, as well as to assist in the definitions of flows to increase the efficiency of services.
